# Predictors of non-compliance with post-procedure follow-up after endoscopic stent placement

**DOI:** 10.1055/a-2760-6670

**Published:** 2026-01-20

**Authors:** Irene C. Perez, Augustin Attwell

**Affiliations:** 1 129263 Gastroenterology and Hepatology, University of Colorado Anschutz Medical Campus, Aurora, United States; 2 2920 Denver Health Medical Center, Denver, United States

**Keywords:** Pancreatobiliary (ERCP/PTCD), Stones, Strictures, Endoscopic ultrasonography, Intervention EUS

## Abstract

**Background and study aims:**

Delay or absence of follow-up after luminal or pancreatobiliary stent placement can lead to adverse events. Few studies have investigated patient factors that impact compliance. The aim of this study was to identify patient-related predictors of compliance and non-compliance for luminal or pancreatobiliary stent removal at a single center.

**Patients and methods:**

Patients who underwent esophagogastroduodenoscopy, endoscopic ultrasound, and/or endoscopic retrograde cholangiopancreatography with temporary stent placement for disease from March 2020 to March 2024 were included. Compliance was defined as stent removal or imaging confirming stent passage within 6 months (plastic stents or any cystgastrostomy stents) or 12 months (metal biliary stents) of the index procedure. Social and demographic risk factors potentially associated with stent removal and non-compliance were analyzed.

**Results:**

One hundred fifty-one cases fit the inclusion criteria, of which 118 involved compliant patients (78%) and 33 (22%) involved non-compliant patients. Time to stent removal was 57 ± 43 days in the compliant group and 324 ± 156 days in the non-compliant group ( *P* < 0.001). Common procedure indications included pancreatitis-related complications (n = 61), biliary obstruction (n = 55), and bile leak (n = 35). Predictors of non-compliance included male sex (0.047), history of drug use (P = 0.033), and absence of a working phone number ( *P* = 0.017) or email address ( *P* = 0.003), electronic medical record access ( *P* < 0.001), or primary care provider ( *P* = 0.034) before the procedure.

**Conclusions:**

Patient-specific risk factors for non-compliance of stent removal were identified. Patients with such risk factors may require extra education and communication efforts.

## Introduction

A delay in luminal or pancreaticobiliary (PB) stent removal can lead to long-term adverse events (AEs), including symptomatic stent occlusion or migration, cholangitis, perforation, recurrent stones, and/or need for surgical intervention 1 2 3 . Procedure non-compliance may also lead to wasted resources, longer procedure wait times, patient dissatisfaction, and missed diagnosis of malignancy. To date, few studies have identified demographic and socioeconomic factors associated with patient compliance regarding follow-up stent management.

Because few studies have identified predictive factors for compliance specifically with PB stent removal, it is unclear if these factors apply to our patient population or others. The goal of this study was to identify demographic, social, and behavioral factors that may predict non-compliance to follow-up after stent insertion at a single, academic safety-net hospital.

## Patients and methods

### Primary and secondary outcomes

The primary outcome was identifying predictors of compliance or non-compliance based on demographic, social behavior, and social determinants of health factors. Demographic factors investigated were age, gender, race and ethnicity, marital status, and preferred language. For social behavior, alcohol or drug use factors were assessed, and for social determinants of health, employment status, homelessness, access to a telephone or electronic health records, active insurance, having a primary care physician (PCP) and appointment with a PCP or gastroenterology specialist within a year prior to index procedure was assessed. The secondary outcome was the rate of AEs attributed to delayed stent removal.

### Study design

The study was approved for exemption by the Sponsored Programs and Research Office (Cayuse 23–0133) and the Colorado Multiple Institutional Review Board (Protocol 23–0780) on April 30, 2023. Cases were identified by current procedural terminology (CPT) codes. Index procedures included esophagogastroduodenoscopy (EGD) with stent placement, endoscopic retrograde cholangiopancreatography (ERCP) with stent placement, and upper endoscopic ultrasound (EUS) with stent placement. Follow-up procedures included ERCP with stent removal and EGD with stent removal. The electronic medical record (EMR) was used to extract data on the different patient factors assessed using codes that were manually verified. The only data extracted manually were having a clinic visit with a gastroenterologist or hepatologist 1 year prior to index procedure and AEs that were collected by chart review. The data search was limited to procedures and imaging at Denver Health Medical Center and select other local and regional hospitals whose information is available on the primary EMR.

The study included all stents—plastic or covered metal—placed for temporary treatment of benign disease including pancreatobiliary (PB) strictures and cystgastrostomy tracts. Plastic biliary and pancreatic stents used for therapeutic purposes were internally flanged or single pigtailed, straight stents (Boston Scientific, Marlborough, Massachusetts, United States). Covered metal stents used for treatment of benign biliary strictures were unflanged, straight Wallflex stents (Boston Scientific). Covered metal lumen-apposing stents (LAMS) (AXIOS, Boston Scientific) and/or double pigtail stents (Boston Scientific) were used for pancreatic pseudocysts and/or organized necrosis. Pancreatic plastic stents (PPS) used for prophylaxis against post-ERCP pancreatitis (PEP) were straight, 4F or 5F, internally flangeless stents with an external single-pigtail or double flanges. All endoscopic procedures were completed by one advanced endoscopist and were conducted with either monitored anesthesia care or general anesthesia.

After the index procedure, patients were contacted by telephone and/or scheduled to undergo follow-up imaging and/or endoscopic procedures for stent removal as detailed below, and at least two attempts were made to schedule these procedures/tests. The interval for scheduling follow-up was as follows: for PBS stent placement, follow-up ERCP in 6 to 12 weeks; for covered metal biliary stent placement, follow-up ERCP in 6 weeks; for LAMS used to drain pseudocyst/necroma, follow-up EGD in 4 to 6 weeks; for prophylactic plastic pancreatic stents, abdominal x-ray in 2 to 4 weeks and selective EGD as soon as possible thereafter if x-ray showed a stent still present.

### Setting

This study was conducted solely at Denver Health Medical Center. Procedures occurred between March 2020-March 2024.

### Inclusion criteria

Patients who underwent endoscopic stent placement into biliary and/or pancreatic ducts, or pseudocysts/necromas for pancreatitis-related complications, biliary obstruction or bile leak, respectively, were included.

### Exclusion criteria

Patients with placement of an uncovered metal stent, unsuccessful stent placement, stent removed during surgery or the index hospitalization, history of a previous stent placement within a year, a stent/s placed for indefinite or permanent duration based on severe medical illness or in the setting of malignancy, and patient death or transition to palliative care within 1 year from index procedure were excluded. Colonoscopy and lower EUS procedures were also excluded.

### Definitions

Compliance was defined as stent removal within 6 months of the index procedure for therapeutic PB stents; within 1 year for metal biliary stents; within 6 months for plastic and metal stents for therapy of pancreatic pseudocyst/necroma; and abdominal imaging showing stent passage or endoscopic removal within 6 months for prophylactic pancreatic duct stents.

Non-compliance was defined as failure to schedule or show up for a follow-up procedure for stent removal or abdominal imaging to confirm stent passage (for prophylactic pancreatic duct stents) within the allotted time.

For specific compliance-related risk factors: alcohol use was defined as any use of alcohol, including previous use, at time of procedure; drug use was defined as any previous or active use; homelessness was defined as having a prior or present history of homelessness in the EMR; telephone was defined as having a listed phone number in the EMR; access to electronic health records was defined as having a registered account in the EMR; having a PCP visit was defined as seeing the PCP in clinic within 1 year prior to the index procedure; and having a gastroenterology specialist visit was defined as seeing a Denver Health gastroenterologist or hepatologist within 1 year prior to index procedure.

### Statistical analysis

Descriptive measures including means, standard deviation, and percentages were used to describe demographic, social behavior, and social determinants of health. Two-sided independent samples test was used for statistical analysis of continuous variables and two-sided Fisher’s exact test or Pearson’s chi-squared test was used for statistical analysis to identify predictors of non-compliance. IBM SPSS Statistics (Version 29.0.2.0 4 ) was used. Complications from delayed stent removal were also noted.

## Results

**Fig. 1:**
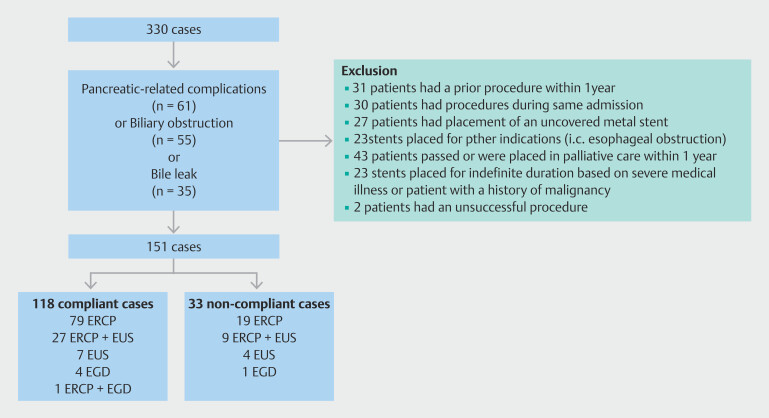
Scheme of identified cases. Index procedures and follow-up cases were identified by CPT codes. Indications for stent placement included pancreatitis-related complications (n = 61), biliary obstruction (n = 55), or biliary leak (n = 35). One hundred fifty-one cases fit the inclusion criteria. Procedures performed for compliant and non-compliant patients are shown.

One hundred fifty-one cases met the inclusion criteria ( Fig. 1 ). One hundred eighteen patients (78%) were categorized as compliant and 33 (22%) as non-compliant. Common indications for stent placement included treatment of symptomatic acute and/or chronic pancreatitis including strictures and pseudocyst/necroma formation (N = 49 compliant and N = 12 non-compliant), biliary obstruction (N = 42 compliant and N = 13 non-compliant), and biliary leak (N = 27 compliant and N = 8 non-compliant) ( Table 1 ). Eleven of the total cases also involved pancreatic stenting for PEP prophylaxis (compliant N = 7 and non-compliant N = 4). There was no association between the indication for a procedure and non-compliance ( *P* = 0.864). Index procedures were mainly performed in the inpatient setting (72%), including 83 cases (70%) in the compliant group and 26 cases (79%) in the non-compliant group ( *P* = 0.338). Follow-up procedures were mainly completed in the outpatient setting with 99 (81%) and 10 (83%) cases in the compliant and non-compliant group, respectively ( *P* = 1.000). Time to stent removal was 57 ± 43 days in the compliant group and 324 ± 156 days in the non-compliant group ( *P* < 0.001). Type of stent used in each group is shown in Table 2 .

**Table:** **Table 1** Procedure indication.

	Compliant (n = 118)	Non-compliant (n = 33)	*P* value
Pancreatitis-related complications (n, %)	49 (40)	12 (37)	0.864
Biliary obstruction (n, %)	42 (36)	13 (39)	
Bile leak (n, %)	27 (22)	8 (24)	
The procedure indication is listed in this table for the compliant and non-compliant groups. Indications for stent placement were pancreatitis-related complications, biliary obstruction, and bile leak.			

**Table:** **Table 2** Stent type used.

	Compliant (n = 118)	Non-compliant (n = 33)	*P* value
Plastic biliary and/or pancreatic stent/s (n, %)	72 (61)	19 (58)	0.936
Metal biliary with/without plastic biliary and/or pancreatic stent/s (n, %)	30 (24)	9 (27)	
Metal and/or plastic cystgastrostomy stent/s with/without plastic pancreatic stent (n, %)	16 (13)	5 (15)	
The type of stent (plastic or metal) used for a procedure is listed in this table for the compliant and non-compliant groups. Stent type categories were divided into plastic biliary and/or pancreatic stent/s, metal biliary with or without plastic biliary and/or pancreatic stent/s, and metal and/or plastic cystgastrostomy stent/s with or without plastic pancreatic stent.			

Mean age of patients was 53.17 ± 16.44 (compliant) and 52.33 ± 16.62 years (non-compliant) ( *P* = 0.797), and there was no association between being under or over 50 years and non-compliance ( *P* = 0.422). Eighty-eight patients (58%) in the entire cohort were male, including 63 (54%) in the compliant group and 24 (73%) in the non-compliance group. Statistical analysis demonstrated that there was a statistically higher number of males in the non-compliance group ( *P* = 0.047). Race and ethnicity were not associated with non-compliance, as shown in Table 3 .

**Table:** **Table 3** Variables related to patient demographics, social determinants of health, and behaviors.

	Compliant	Non-compliant	*P* value
Age (n, %)	n = 118	n = 33	
> 50 years	70 (59)	17 (52)	0.422
Sex (n, %)	N = 118	N = 33	
Male	63 (54)	24 (73)	0.047*
Female	57 (46)	9 (27)	
Race (n, %)	N = 115	N = 32	
White	64 (56)	19 (59)	0.707
Non-White	51 (44)	13 (41)	
Ethnicity (n, %)	N = 115	N = 32	
Non-Hispanic	62 (54)	12 (38)	0.100
Hispanic	53 (46)	20 (63)	
Marital status (n, %)	N = 118	N = 33	
Single, divorced or widowed	79 (67)	26 (79)	0.191
Married or partner	39 (33)	7 (21)	
Language (n, %)	N = 118	N = 33	
English	78 (66)	22 (67)	0.952
Non-English	40 (34)	11 (33)	
Employment (n, %)	N = 117	N = 33	
Yes	27 (23)	4 (12)	0.170
Disabled (n, %)	N = 118	N = 33	
Yes	21 (18)	8 (24)	0.406
Homelessness (n, %)	N = 118	N = 33	
Yes	3 (3)	3 (9)	0.119
Insurance (n, %)	N = 118	N = 33	
No	22 (19)	8 (24)	0.714
Private	23 (20)	7 (21)	
Non-private	73 (62)	18 (55)	
Drug use (n, %)	N = 118	N = 33	
Yes	37 (31)	17 (52)	0.033*
Alcohol use (n, %)	N = 118	N = 33	
Yes	62 (53)	21 (64)	0.257
Phone (n, %)	N = 118	N = 33	
Yes	66 (56)	26 (79)	0.017*
Email address (n, %)	N = 118	N = 33	
Yes	92 (78)	20 (51)	0.003*
EMR participation (n, %)	N = 118	N = 33	
Yes	88 (75)	14 (42)	<0.001*
PCP (n, %)	N = 118	N = 33	
Yes	90 (76)	19 (58)	0.034*
PCP visit (n, %)	N = 118	N = 33	
Yes	65 (55)	12 (36)	0.057
Gastroenterology visit (n, %)	N = 118	N = 33	
Yes	26 (22)	5 (15)	0.387
PCP, primary care physician. Demographic, social behavior and social determinants of health factors that were assessed as predictors of compliance or non-compliance to stent removal are listed in this table with associated *P* values. Predictors of non-compliance were male sex, previous or active drug use, lack of access to a working phone, email address, electronic medical record, or primary care provider. * p < 0.05			

The preferred language was English in both groups with 78 (66%) and 22 (67%) of patients in the compliant and non-compliant groups, respectively ( *P* = 0.952). Primary non-English languages spoken included Spanish, Arabic, Vietnamese, Polish, Cantonese, Mandarin and Armenian. Of those that spoke Spanish there were 31 of 118 (26%) and 10 of 33 (30%) patients in the compliant and non-compliant groups, respectively ( *P* = 0.645). Most patients in both groups were single (never married, divorced, or widowed) including 79 (67%) in the compliant group and 26 (79%) in the non-compliant group, and the remainder were married or with a partner including 39 (33%) in the compliant group and seven (21%) in the non-compliant group ( *P* = 0.191). In the compliant group, 96 patients (81%) were insured, including 23 (20%) with private insurance and 73 (62%) with Medicaid or Medicare, and of the non-compliant group, 25 (76%) patients were insured, including seven (21%) with private insurance and 18 (55%) with Medicaid/Medicare ( *P* = 0.714). Preferred language, marital status, and insurance status were not predictors of non-compliance ( Table 1 ).

Prevalence of employment was low in both groups, 27 (23%) in the compliant group compared with four (12%) in the non-compliant group ( *P* = 0.170). Rates of disability and homelessness in each group are shown in Table 3 . All these factors were non-predictive of compliance. Drug use was statistically more prevalent in the non-compliant patients N = 17 (52%) versus compliant patients N = 37 (32%) ( *P* = 0.033), and any alcohol use was higher in the non-compliant group but was not statistically significant (compliant N = 62 (53%) vs non-compliant N = 21 (64%) ( *P* = 0.257) ( Table 3 ).

Patients without a working phone number, email address, or access to the EMR were associated with non-compliance: 66 patients (56%) vs 26 (79%) had a phone ( *P* = 0.017); 92 (78%) vs 20 (51%) patients had an email address ( *P* = 0.003); and 88 (75%) patients vs 14 (42%) patients had access to the EMR in the compliance and non-compliant groups, respectively ( *P* < 0.001). Finally, presence of a PCP was statistically associated with compliance: having a PCP, 90 compliant patients (76%) vs 19 non-compliant patients (58%) ( *P* = 0.034). Having a PCP or gastroenterology appointment in the year prior to the procedure was not statistically different among the two groups as shown in Table 3 .

There were two AEs related to delayed stent removal. One patient with a common bile duct stent placed for a bile leak and a ventral pancreatic duct stent placed for prophylactic PEP developed obstructive jaundice with septic shock and *Escherichia coli* bacteremia and was successfully treated medically. Another patient who had a stent placed for a bile leak presented with nausea, vomiting and abdominal pain with labs concerning for biliary obstruction. The patient’s symptoms resolved after stent removal.

## Discussion

In this 4-year retrospective study from a safety-net hospital analyzing 151 patients undergoing luminal or PB stent placement, 33 patients (22%) were found to have delayed or absent stent removal. Compared with the few other studies (inside and outside the United States) reporting rates of 5% to 19% for delayed or absent stent removal/change after ERCP, the non-compliance rate in this study is higher and is as expected in a safety-net hospital patient population 5 6 . In our study, male sex, a non-working phone, substance use, lack of access to email or EMR, and no affiliation with a PCP were predictors of non-compliance. In addition, a low number of AEs was found in patients with delayed stent removal (2/33 [6%]).

For general gastrointestinal endoscopy, no-show rates vary according to practice setting but range from 12% to 42% 7 8 9 . Factors associated with non-compliance to follow-up include prior non-compliance, opioid or benzodiazepine use, substance abuse, homelessness, unemployment, non-private insurance, and a procedure wait time of greater than 2 months 7 10 11 . In one study, older age (71.23 ± 12.17 years) and miscommunication with the medical team were associated with delayed biliary stent removal 5 6 . Non-English speakers and patients requiring anesthesia support for ERCP have also been associated with delayed biliary stent removal 6 . In addition, in a safety-net hospital, male sex, African-American race, single marital status, and lack of private insurance were associated with non-compliance to follow-up general and advanced gastrointestinal procedures including ERCP and EUS 12 . In this study, male sex was associated with non-compliance; however, a non-English speaker, race, marital status, and lack of insurance were not associated with non-compliance. It has also been found that patients undergoing advanced procedures and attending a pre-procedure appointment are more likely to show up for a scheduled procedure 8 10 13 .

In terms of timing for stent removal/exchange, for benign biliary strictures, PBS should be exchanged or upsized every 3 to 4 months for up to 12 months and covered metal biliary stents should be removed within 6 months 14 15 . For a bile or pancreatic duct injury, PBS or PPS removal is typically done within 6 to 8 weeks 14 15 . After ERCP, prophylactic PPS should be removed within 4 weeks if imaging does not show spontaneous passage 16 . For refractory pancreatic duct strictures, timing of stent exchange is variable, although replacement is typically needed within 3 to 6 months 14 . For pancreatic pseudocyst or walled of necrosis, lumen-apposing metal stents and plastic double pigtail stents are used through the treatment period, which is highly variable, but stent change or removal is typically less than 3 months depending on various factors such as degree of necrosis, size, and need for necrosectomy 17 18 .

To our knowledge, this is the first study evaluating various social determinants of health and behaviors as predictors of non-compliance for advanced endoscopic procedures. Similar to the study by Chang et al., we found that a history of substance use was associated with non-compliance 10 . In addition, patients with additional forms of hospital communication beyond phone calls (e.g. email, direct messaging via the EMR), would be expected to have higher compliance, and this trend is supported by our study. However, it is unclear if this communication advantage stems from medical literacy or just having an additional reminder. Finally, the relationship between having a PCP and procedure compliance is unclear. We suspect that having a physician relationship pre-selects patients more likely to be motivated and organized about their health care in general, and extra physician appointments can simply serve as reminders for upcoming procedures.

Future strategies to improve procedure compliance and efficiency may include overbooking scheduled procedures for high-risk patients and spending more time/effort explaining the importance of stent follow-up and dangers of stent retention to patients with non-compliance risk factors. Physicians may also need to modify or highlight the follow-up in any procedure discharge instructions. Patients may also benefit from pre-procedure reminder phone calls, which is a routine part of our pre-procedure care. Gu et al. showed that monthly reminders via mobile phone increased compliance with PBS removal 19 . In another study, implementation of phone call reminders by a nurse 7 days prior to a scheduled procedure was shown to improve compliance for general gastrointestinal endoscopy 20 .

Alternative strategies could involve enhancing patient involvement in the healthcare system, such as referring them to primary care if they lack a PCP, routing the procedure note to the PCP, and referring the patient to a case manager. The after-visit summary including planned follow-up should also be reviewed with the patient and designated chaperone after the procedure. Furthermore, the European Society of Gastrointestinal Endoscopy recommends maintaining a list of patients with indwelling biliary stents to track those needing reminders for stent removal or exchange, and this may be an efficient strategy to consider 15 .

In the future, use of biodegradable stents that may avoid endoscopic follow-up may be an option for patients. The braided PDX biliary stent and the ARCHIMEDES stent have been shown, in a few studies, to be safe and have good efficacy for postoperative cystic duct leaks, benign biliary strictures, PEP prophylaxis and complications from chronic pancreatitis. Technical and clinical success rates of 94% to 100% and 78% to 90% have been reported 4 21 22 . The rate of premature stent migration is variable with one study reporting the highest rate observed at 9% 4 21 23 . Larger prospective studies are needed before these stents are used in routine practice in the United States.

There are several limitations to this study. One limitation is its retrospective nature with possible confounding variables, including longer waitlist times during the COVID-19 pandemic, which may have impacted patient compliance (125 patients [83%]) in the study underwent index procedures during the pandemic). To mitigate variance in waiting times during the 4 years of the study, we selected the relatively generous 6- and 12-month cut-offs for therapeutic stent removal to define compliance. Presence or absence of stent-related symptoms at time of stent removal would be clinically relevant and interesting; however, such data could not be captured because of the study design, i.e. use of CPT codes. This study was also done at a single medical center, which yielded a relatively small number of cases, raising the possibility of type II statistical errors.

Based on the study design, we were also unable to quantify specific alcohol or drug use and could not determine patient education level. Because presence of substance or alcohol use was based on patient self-reporting, and alcohol consumption or drug use was defined as any present or previous use at time of the index procedure, it is possible that some patients with these risk factors could be missed or miscategorized. It is also possible that some patients could have moved, died, or undergone stent removal at another hospital, which could have been missed by the data search. Although uncommon, loss of follow-up could also contribute to the low AE rate. To help mitigate these confounding factors, we expanded our search of follow-up procedures to include procedures completed at local and regional hospitals available in the EMR system. Given that our resource-limited patients generally restrict their outpatient care to our center and outside physicians typically communicate or transfer the patients back, we believe the impact on our database to be minimal. Despite these limitations, we opted to include patients without any follow-up and place them in the non-compliant group. Finally, because the study only included patients at a safety-net hospital, generalizability to larger or non-US centers may be limited.

## Conclusions

Compared with general gastrointestinal endoscopy, advanced endoscopic procedures can lead to higher morbidity and mortality, particularly if stents are not changed or removed appropriately. This study identifies patient variables that may be used to risk stratify a patient as non-compliant and to guide development of interventions to increase compliance. Larger, multicenter studies are needed to confirm our findings. Until then, additional measures to improve compliance in patients with identifiable risk factors may lessen patient morbidity and improve endoscopic efficiency.
